# Galactose‐modified duocarmycin prodrugs as senolytics

**DOI:** 10.1111/acel.13133

**Published:** 2020-03-16

**Authors:** Ana Guerrero, Romain Guiho, Nicolás Herranz, Anthony Uren, Dominic J. Withers, Juan Pedro Martínez‐Barbera, Lutz F. Tietze, Jesús Gil

**Affiliations:** ^1^ MRC London Institute of Medical Sciences (LMS) London UK; ^2^ Faculty of Medicine Institute of Clinical Sciences (ICS) Imperial College London London UK; ^3^ Developmental Biology and Cancer Programme Birth Defects Research Centre Great Ormond Street Institute of Child Health University College London London UK; ^4^ Institute of Organic and Biomolecular Chemistry Georg‐August University Göttingen Germany

**Keywords:** duocarmycin, prodrug, senescence, senescence‐associated β‐galactosidase, senolytics

## Abstract

Senescence is a stable growth arrest that impairs the replication of damaged, old or preneoplastic cells, therefore contributing to tissue homeostasis. Senescent cells accumulate during ageing and are associated with cancer, fibrosis and many age‐related pathologies. Recent evidence suggests that the selective elimination of senescent cells can be effective on the treatment of many of these senescence‐associated diseases. A universal characteristic of senescent cells is that they display elevated activity of the lysosomal β‐galactosidase, and this has been exploited as a marker for senescence (senescence‐associated β‐galactosidase activity). Consequently, we hypothesized that galactose‐modified cytotoxic prodrugs will be preferentially processed by senescent cells, resulting in their selective killing. Here, we show that different galactose‐modified duocarmycin (GMD) derivatives preferentially kill senescent cells. GMD prodrugs induce selective apoptosis of senescent cells in a lysosomal β‐galactosidase (GLB1)‐dependent manner. GMD prodrugs can eliminate a broad range of senescent cells in culture, and treatment with a GMD prodrug enhances the elimination of bystander senescent cells that accumulate upon whole‐body irradiation treatment of mice. Moreover, taking advantage of a mouse model of adamantinomatous craniopharyngioma (ACP), we show that treatment with a GMD prodrug selectively reduced the number of β‐catenin‐positive preneoplastic senescent cells. In summary, the above results make a case for testing the potential of galactose‐modified duocarmycin prodrugs to treat senescence‐related pathologies.

## Graphical abstract

Galactose‐modified duocarmycin prodrugs get preferentially processed in senescent cells and behave as senolytics.

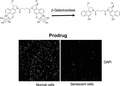

## 1 INTRODUCTION

Cellular senescence is a stress response that prevents the replication of old, damaged or transformed cells (Herranz & Gil, 2018). Senescence can be induced by replicative exhaustion and also by a range of insults that includes oncogenic activation, genotoxic stress or irradiation. The defining feature of senescence is a stable cell cycle arrest, but senescent cells also undergo multiple phenotypic changes including alterations in their morphology, metabolic state or chromatin arrangement (Salama, Sadaie, Hoare, & Narita, 2014). In particular, senescent cells secrete a combination of extracellular factors, the so‐called senescence‐associated secretory phenotype or SASP, which is a prominent mediator of the patho‐physiological effects of senescence (Acosta et al., 2008, 2013; Coppe, Desprez, Krtolica, & Campisi, 2010; Kuilman & Peeper, 2009).

Despite that the acute induction of senescence limits fibrosis and protects against cancer progression, the abnormal accumulation of senescent cells with age or in diseased tissues is detrimental (Munoz‐Espin & Serrano, 2014). Interestingly, evidence drawn from genetic models has shown that eliminating senescent cells increases lifespan, improves healthspan and benefits the outcomes of a wide range of diseases (Baker et al., 2011, 2016; Childs et al., 2016, 2017). These studies have led to a collective effort to identify “senolytics,” drugs that selectively kill senescent cells. Several senolytics have been identified including dasatinib and quercetin (Zhu et al., 2015), piperlongumine (Wang et al., 2016), FOXO4‐interfering peptides (Baar et al., 2017), HSP90 inhibitors (Fuhrmann‐Stroissnigg et al., 2017), cardiac glycosides (Guerrero et al., 2019, 2015; Triana‐Martinez et al., 2019) or the Bcl2 family inhibitors ABT‐263 (navitoclax) and ABT‐737 (Chen et al., 2015; Yosef et al., 2016; Zhu et al., 2016). Currently, Bcl2 family inhibitors have become the gold standard on senolysis. Bcl2 family inhibitors eliminate a range of senescent cells in vivo and reproduce the effects observed in transgenic mice modelling senescence ablation (Ovadya & Krizhanovsky, 2018). However, ABT‐263 causes severe thrombocytopenia and neutropenia, what might complicate its use on the clinic. Moreover, it is becoming evident that specific senolytics might be necessary to eliminate different types of senescent cells. Therefore, there is a need to identify additional drugs with senolytic properties.

An alternative strategy for targeting senescence is to exploit properties that differentiate senescent from normal cells. In this regard, the senescence‐associated β‐galactosidase activity (SA‐β‐gal) is one of the more conserved and defining characteristics of senescent cells. Senescent cells present an increased lysosomal mass (Kurz, Decary, Hong, & Erusalimsky, 2000). As a result, senescent cells display elevated levels of lysosomal enzymes such as β‐galactosidase (encoded by *GLB1* (Dimri et al., 1995)) or α‐fucosidases (Hildebrand et al., 2013). Indeed, it has been shown that galacto‐oligosaccharide encapsulated nanoparticles (GalNP) preferentially release their content on senescent cells (Agostini et al., 2012). Consequently, this GalNP can be used in combination with different cargos to either image or kill senescent cells (Munoz‐Espin et al., 2018).

Galactose modification has been frequently used to improve the pharmacokinetic properties or the delivery of existing drugs. In addition, galactose modification can be used to generate prodrugs that rely on *E. coli* β‐galactosidase for controlled activation (Melisi, Curcio, Luongo, Morelli, & Rimoli, 2011). When combined with antibody‐linked β‐galactosidase, this approach is known as antibody‐directed enzyme prodrug therapy (ADEPT) (Bagshawe, 2006; Tietze & Schmuck, 2011). In ADEPT, a conjugate of a tumour‐specific antibody and an enzyme, such as β‐galactosidase, is combined with the application of a hardly cytotoxic prodrug. By means of the enzyme in the conjugate, the prodrug is selectively cleaved in cancer cells leading to the formation of a highly cytotoxic compound. Several of these galactose‐modified cytotoxic prodrugs have been described (Leenders et al., 1999). A class of such prodrugs are galactose‐modified duocarmycin (GMD) derivatives (Tietze, Major, & Schuberth, 2006). Duocarmycins are a group of antineoplastic agents with low picomolar potency. They are thought to act by binding and alkylating double‐stranded DNA in AT‐rich regions of the minor groove (Boger, Johnson, & Yun, 1994; Tietze et al., 2006; Tietze, Schuster, Krewer, & Schuberth, 2009), but alternative mechanisms of action have been proposed to account for the cytotoxic effects of duocarmycin dimers (Wirth, Schmuck, Tietze, & Sieber, 2012).

Here, we investigated whether galactose‐modified prodrugs can preferentially kill senescent cells. We have assessed several GMD derivatives and confirmed their senolytic potential in cell culture, ex vivo and in vivo. Given the increasing list of senescence‐associated diseases and the benefits of senolytic treatment, we propose that GMD derivatives and, more generally, galactose‐modified prodrugs are a new class of senolytic compounds and they should be tested to assess their therapeutic potential.

## 2 RESULTS

### 2.1 A galactose‐modified duocarmycin prodrug with senolytic properties

**Figure 1:**
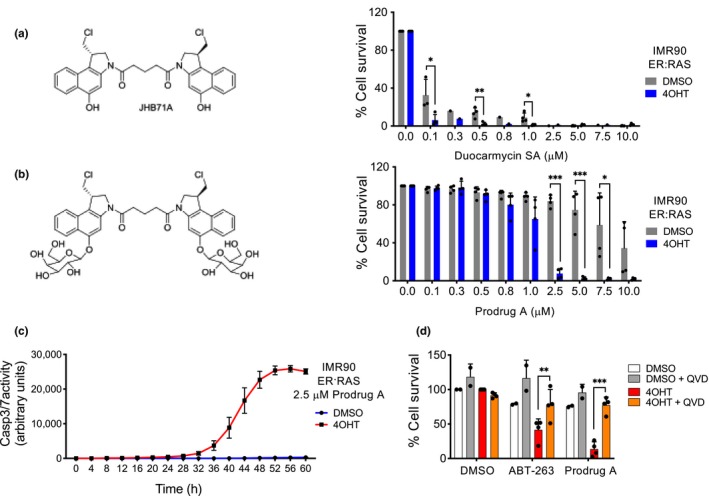
A galactose‐modified duocarmycin prodrug with senolytic properties. (a) Molecular structure of seco‐duocarmycin analog dimer (JHB71A, left). IMR90 ER:RAS cells were treated with DMSO or with 4OHT (4‐hydroxy‐tamoxifen) for 6 days to induce OIS. Cells were treated with the indicated concentrations of seco‐duocarmycin analog dimer for 72 hr. Cell numbers were quantified using DAPI staining, and percentage of survival cells are plotted (right) (*n* = 4). (b) Molecular structure of a galactose‐modified prodrug derivative of seco‐duocarmycin analog dimer (JHB75B, referred as prodrug A, left). Cells were treated with prodrug A for 72 hr as described before (*n* = 4). (c) Treatment of senescent cells with a GMD prodrug triggers caspase‐3/7 activity. IMR90 ER:RAS were treated with 4OHT or vehicle (DMSO) for 6 days to induce senescence. 2.5 μM prodrug was then added together with NucLight Rapid Red Reagent for cell labelling and Caspase‐3/7 reagent for apoptosis (IncuCyte). Caspase 3/7 activity was measured at 4‐hr intervals. (d) After 6‐day treatment with 4OHT or vehicle (DMSO), IMR90 ER:RAS were treated with 1 μM ABT‐263 or 2.5 μM prodrug A for 72 hr in the presence or absence of the pan‐caspase inhibitor Q‐VD‐OPh (*n* = 4). All statistical significances were calculated using unpaired Student's *t* tests. All error bars represent mean ± s.d; *n* represents independent experiments.; ns, not significant; *p < .05; **p < .01; ***p < .001, ****p < .0001

The natural antibiotic duocarmycin is a highly cytostatic compound (Boger & Johnson, 1995). A series of glycosidic derivatives of duocarmycin have been previously developed to be used as prodrugs in the context of antibody‐directed enzyme prodrug therapy (ADEPT) (Tietze, Hof, Muller, Krewer, & Schuberth, 2010; Tietze et al., 2009). Given that senescent cells display elevated levels of SA‐β‐galactosidase activity, we hypothesized that galactose‐modified cytotoxic prodrugs will be preferentially processed by senescent cells, resulting in their selective killing. To test this hypothesis, we took advantage of a galactose‐modified duocarmycin (GMD) prodrug (referred as prodrug A, JHB75B) previously described (Tietze et al., 2009). We analysed the effects that a seco‐duocarmycin analogue dimer (duocarmycin SA) and its galactose derivative (prodrug A) had on the survival of IMR90 ER:RAS cells, a model of oncogene‐induced senescence (OIS). Activation of the ER:RAS fusion with 4‐hydroxy‐tamoxifen (4OHT) induces senescence in IMR90 ER:RAS cells (Georgilis et al., 2018). Treatment with duocarmycin SA was equally effective in killing normal and senescent cells, with the exception of a small selectivity towards senescent cells at the lower concentrations (Figure 1a). In contrast, when we treated IMR90 ER:RAS cells with prodrug A (differing only in the addition of two galactose groups that inactivate it), we observed the preferential elimination of senescent cells (Figure 1b and Figure S1a). Duocarmycins are known to bind and alkylate DNA in AT‐rich regions of the minor groove and induce cell death in a way dependent of DNA replication (Boger et al., 1994; Tietze et al., 2006, 2009) We checked that senescent cells were growth arrested at the time of the drug treatment (Figure S1b). This shows that the effect observed is not due to hyperreplication of cells during early stages of OIS and suggests that the prodrug might act by some of the alternative cytotoxic mechanisms described for duocarmycin dimers (Wirth et al., 2012). Treatment with prodrug A induced caspase 3/7 activity on senescent cells (Figure 1c), and the selective death of these cells was prevented with a pan‐caspase inhibitor (Figure 1d). The above results suggest that GMD prodrugs can behave as senolytics by selectively inducing apoptosis on senescent cells.

### 2.2 **Senolytic properties of prodrug A depend on the lysosomal **β**‐galactosidase**

**Figure 2:**
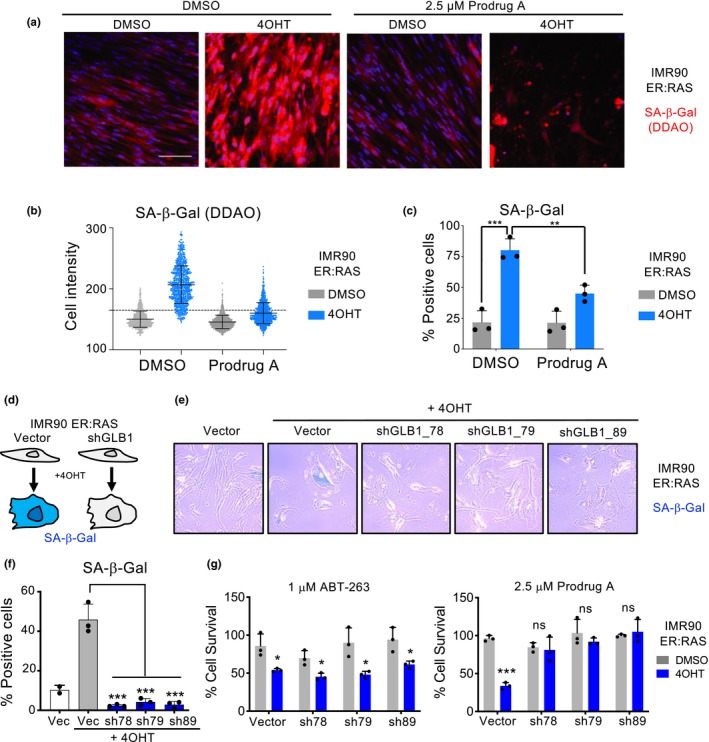
Galactose‐modified duocarmycin prodrugs preferentially target senescent cells with high SA‐β‐galactosidase activity. (a) Representative pictures of fluorescent SA‐β‐galactosidase staining in IMR90 ER:RAS cells treated with prodrug A or vehicle. Scale bar, 100 µm. (b) Single‐cell intensities value for DDAO galactoside in a representative well of a 96‐well plate seeded with IMR90 ER:RAS cells treated with DMSO or 4OHT. Grey dotted line indicates quantification cut‐off. Cells were considered positives for SA‐β‐gal when their cell intensity was > 165. (c) Quantification of SA‐β‐galactosidase activity in IMR90 ER:RAS cells treated with prodrug A or vehicle. Statistical significance was calculated using two‐way ANOVA. (d‐g) The senolytic properties of prodrug A depend on the lysosomal β‐galactosidase (GLB1). (d) Experimental set‐up. (e) Representative pictures of cytochemical SA‐β‐Gal staining in IMR90 ER:RAS infected with different shRNAs against *GLB1* or an empty vector and (f) quantification (*n* = 3). Statistical significance was calculated using one‐way ANOVA. (g) Quantification of cell survival of senescent and control IMR90 ER:RAS infected with different shRNAs targeting *GLB1* or an empty vector and treated with ABT‐263 or prodrug A for 3 days (*n* = 3). Statistical significance was calculated using two‐tailed, Student's *t* test. All error bars represent mean ± *SD*; *n* represents independent experiments; ns, not significant; *p < .05; **p < .01; ***p < .001, ****p < .0001

We had hypothesized that GMD prodrugs could behave as senolytics due to the higher SA‐β‐galactosidase activity of senescent cells. To investigate whether the levels of β‐galactosidase activity correlate with the sensitivity of senescent cells to GMD prodrugs, we induced senescence in IMR90 ER:RAS cells. Afterwards, we treated control or senescent cells with 2.5 µM prodrug A and used a fluorescent substrate (DDAO) to quantify SA‐β‐galactosidase activity at single‐cell resolution (Figure 2a). IMR90 ER:RAS cells undergoing OIS had more cells with higher SA‐β‐galactosidase levels, as it was evident when analysing cell intensities (Figure 2b), or when using threshold system to quantify the percentage of SA‐β‐galactosidase‐positive cells (Figure 2c) or divide the cells in negative, high and highest for SA‐β‐galactosidase (Figure S2). While treatment with 2.5 µM prodrug A selective killed the majority of the senescent cells (Figure S1a), it was interesting to observe that the senescent cells that survived display lower levels of SA‐β‐galactosidase activity (Figure 2a–c and Figure S2), linking the senolytic selectivity of prodrug A with the SA‐β‐galactosidase activity.

The increase in β‐galactosidase observed on senescent cells is due to an increase in lysosomal mass (Kurz et al., 2000) resulting in higher activity of the lysosomal β‐galactosidase (encoded by *GLB1*) (Lee et al., 2006). To further prove that the senolytic activity of GMD prodrugs is dependent on SA‐β‐galactosidase, we took advantage of three independent shRNAs to knock down *GLB1* (Figure 2d). Knock‐down of *GLB1* in IMR90 ER:RAS cells resulted in decreased SA‐β‐galactosidase activity, but it did not impact the growth arrest or the induction of p16^INK4a^ observed during OIS (Figure 2e,f and Figure S3a,b). Taking advantage of these cells, we observed that *GLB1* knock‐down did not affect the senolytic potential of ABT‐263 but ablated the ability of prodrug A to selectively kill senescent cells (Figure 2g and Figure S3c). In summary, our data suggest that GMD prodrugs trigger apoptosis of senescent cells in a GLB1‐dependent manner.

### 2.3 Galactose‐modified duocarmycin prodrugs are broad‐spectrum senolytics

**Figure 3:**
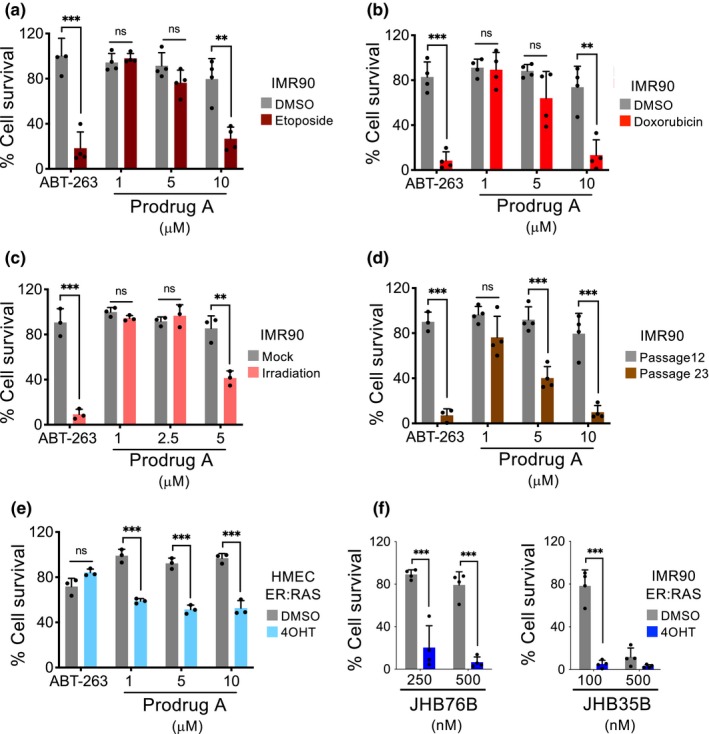
Galactose‐modified duocarmycin prodrugs are broad‐spectrum senolytics. (a–d) Quantification of cell survival after treatment with prodrug A in IMR90 undergoing different types of senescence. Senescence was induced by treatment with 50 μM etoposide (a, *n* = 4), 0.5 μM doxorubicin (b, *n* = 4) or 7.5 Gy irradiation (c, *n* = 3). In (d), the effect of prodrug A on replicative senescence of IMR90 cells (passage 12 versus passage 22) (*n* = 4) was assessed. (e) Quantification of cell survival after treatment with prodrug A in HMEC ER:RAS, human mammary epithelial cells expressing hTERT that undergo senescence upon activation of ER:RAS by 4OHT treatment (*n* = 3). (f) Quantification of cell survival after treatment with two other galactose‐modified duocarmycin derivatives, JHB76B and JHB35B, in the context of oncogene‐induced senescence in IMR90 ER:RAS (*n* = 4). An extended version of this figure including additional drug concentrations is shown in Figure S2. All statistical significances were calculated using unpaired Student's *t* tests. All error bars represent mean ± *SD*; *n* represents independent experiments; ns, not significant; *p < .05; **p < .01; ***p < .001

To understand the extent to which GMD prodrugs behave as senolytics, we assessed the effect that prodrug A has on several types of senescent cells. To this end, we took advantage of IMR90 cells and induced senescence by etoposide or doxorubicin treatment, irradiation, or serial passage (Figure S4a–d). In all those instances, treatment with prodrug A resulted in the selective elimination of senescent cells (Figure 3a–d). Moreover, to evaluate whether the senolytic effects of prodrug A were restricted to IMR90 cells or also observed in other cell types, we took advantage of human mammary epithelial cells able to undergo OIS upon Ras activation (HMEC ER:RAS, Figure S4e). Prodrug A was also able to selectively kill HMEC senescent cells (Figure 3e), suggesting that its senolytic effects were not cell type restricted. Finally, we wanted to examine whether the senolytic properties were specific of prodrug A, or the general concept (conversion of other cytotoxic drugs in galactose‐modified prodrugs) was wider. To this end, we took advantage of two previously described GMD prodrugs, JHB76B and JHB35B (Tietze et al., 2009, 2010). Both drugs were also effective in selectively eliminating senescent cells (Figure 3f and Figure S5), suggesting that the generation of galactose‐modified prodrugs might be a general route to design senolytic compounds.

### 2.4 Prodrug A exerts a bystander effect

**Figure 4:**
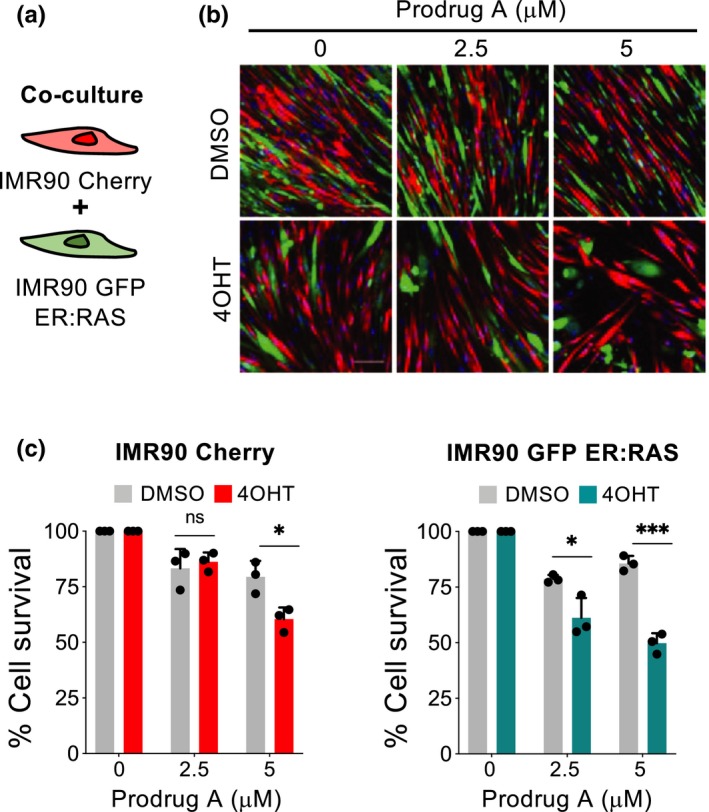
Bystander effect caused by prodrug A in cocultures of normal and senescent cells. (a) Experimental set‐up. (b) Representative pictures of the co‐culture experiment of IMR90 ER:RAS (GFP positive) with wild‐type IMR90 (RFP positive) cells. (c) Quantification of cell survival for each population of the co‐culture experiment after treatment with different concentrations of prodrug A or vehicle (*n* = 3). Statistical significance was calculated using unpaired Student's *t* tests. All error bars represent mean ± s.d; *n* represents independent experiments; ns, not significant; *p < .05; **p < .01; ***p < .001

The above experiments suggest that senescent cells preferentially convert GMD prodrugs into their active duocarmycin derivatives. Since duocarmycins have strong cytostatic properties (Boger & Johnson, 1995), we wonder whether the conversion of GMD prodrugs into their active compounds could result in the bystander killing of normal cells. To assess this, we took advantage of cocultures of normal and senescent cells expressing different fluorescent proteins (Figure 4a). We then treated the cocultures with prodrug A and observed that while lower concentrations killed a subset of senescent cells without affecting to the normal cells, at higher concentrations, a subset of the normal cells also died (Figure 4b,c). Interestingly, a higher percentage of senescent cells survived in cocultures (Figure 4c) that in monocultures (Figure 1b) treated with 5 µM prodrug A, further suggesting the existence of a bystander effect.

### 2.5 Prodrug A eliminates senescent cells in vivo

**Figure 5:**
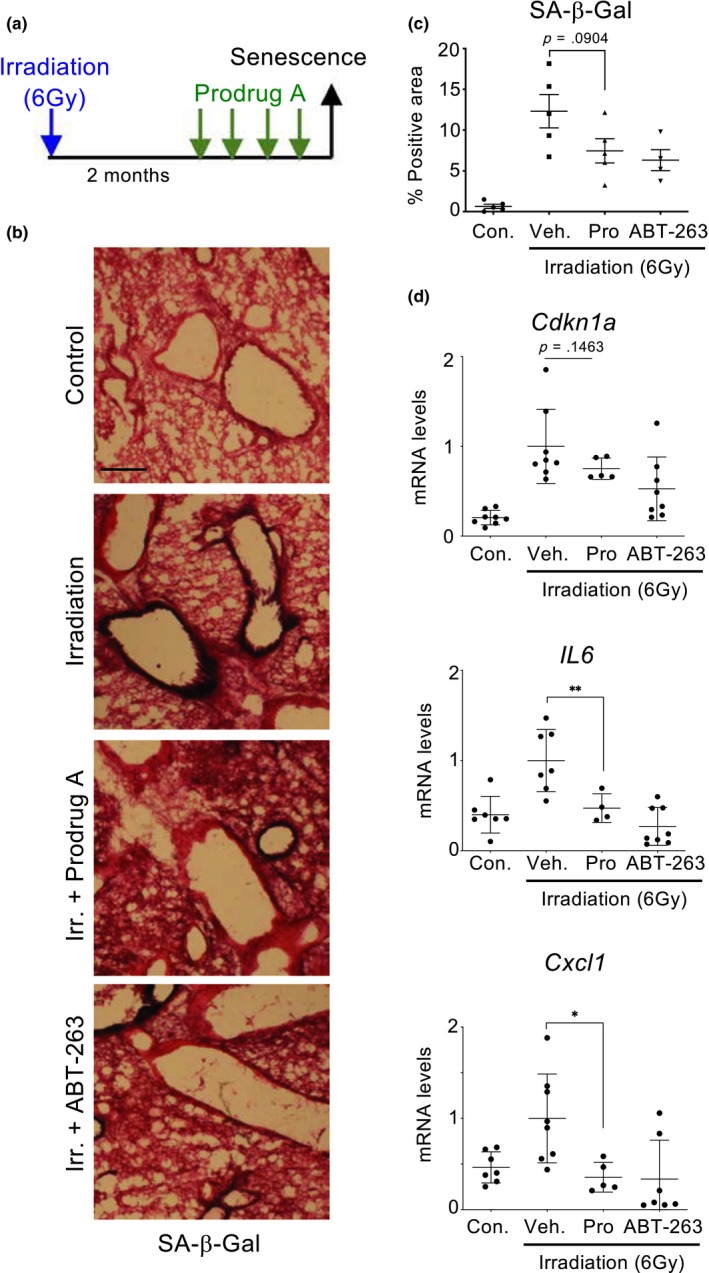
Prodrug A reduces the numbers of senescent cells accumulating after whole‐body irradiation. (a) Experimental design of the whole‐body irradiation‐induced senescence experiment. Mice (*n* = 4/5 per group) were irradiated with 6 Gray to induce senescence. Two months later, mice were treated with vehicle, prodrug A (JHB75B) or ABT‐263 for 4 consecutive days, before being culled for analysis. (b, c) Representative pictures of lung cryosections (b) and quantification of the lung area positive for SA‐β‐Gal staining (c). (d) Expression levels of *Cdkn1a*, *Il6* and *Cxcl1* in lungs of nonirradiated mice or irradiated mice treated with prodrug A, ABT‐263 or vehicle. Statistical significance was calculated using unpaired Student's *t* test. Data represent mean ± *SD*; *n* represents number of mice; ns, not significant; *p < .05; **p < .01

Chemotherapy and radiotherapy are amongst the most common anticancer treatments. Irradiation, chemotherapy and even some targeted anticancer drugs, all induce senescence (Schmitt et al., 2002; Wang et al., 2017). Although induction of tumour senescence explains the anticancer properties of these treatments, the generation of bystander senescent cells is responsible for their side effects (Demaria et al., 2017). To assess whether prodrug A could eliminate these senescent cells, we first irradiated mice and upon a latency period to allow for the accumulation of senescent cells, treated them with prodrug A, ABT263 or vehicle (Figure 5a). Treatment with prodrug A or ABT‐263 resulted in a reduced presence of senescent cells in lung as assessed using SA‐β‐galactosidase activity (Figure 5b,c). Furthermore, a similar trend was observed when we assessed the expression of *Cdkn1a* (that encodes for p21^Cip1^) or the SASP components *Il6* and *Cxcl1* (Figure 5d).

### 2.6 Galactose‐modified prodrugs eliminate preneoplastic senescent cells

**Figure 6:**
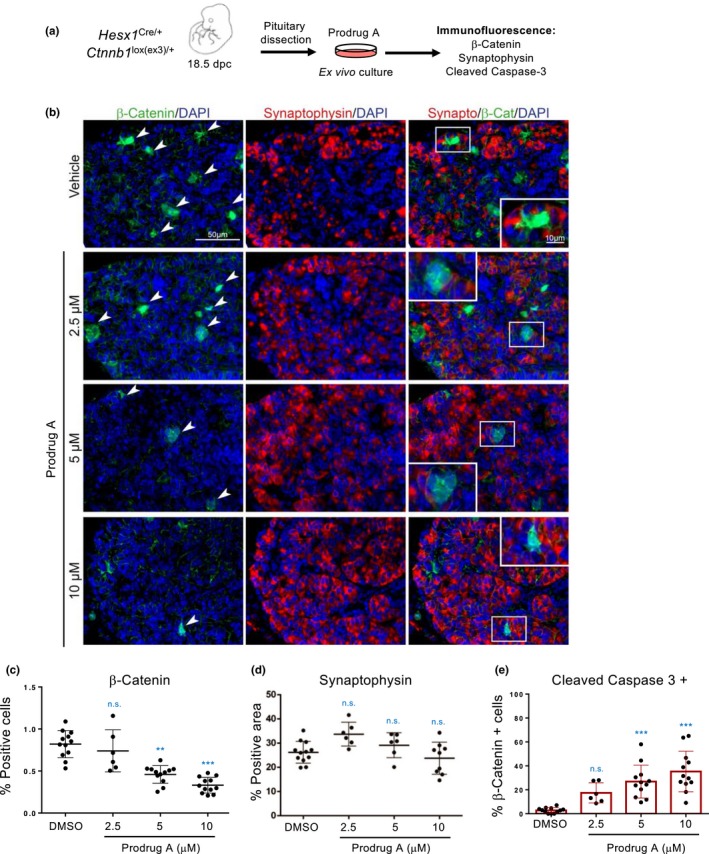
Galactose‐modified duocarmycin prodrug eliminates preneoplastic senescent lesions. (a) Experimental design for the senolytic experiment in the *Hesx1^Cre/+^;Ctnnb1^lox(ex3)/+^* mouse model of adamantinomatous craniopharyngioma (ACP). Tumoural pituitaries from 18.5dpc *Hesx1^Cre/+^;Ctnnb1^lox(ex3)/+^* embryos were cultured in the presence of prodrug A at the indicated concentrations or vehicle (DMSO) and processed for analysis after 72 hr. (b) Immunofluorescence staining against β‐catenin (green) and synaptophysin (red) is shown. Synaptophysin is a marker of the normal hormone‐producing cells in the pituitary gland. Scale bar, 50μm. (c) Quantification of β‐catenin‐accumulating cells after treatment with different concentrations of prodrug A or vehicle (*n* = 6–12). (d) Quantification of synaptophysin‐positive cells after treatment with different concentrations of prodrug A or vehicle (*n* = 6–12). (e) Quantification of β‐catenin‐accumulating cells positive for cleaved caspase‐3 after treatment with different concentrations of prodrug A or vehicle (*n* = 6–12). All statistical significances were calculated using nonparametric ANOVA with Dunn's post hoc comparison. All error bars represent mean ± *SD*; *n* represents number of pituitaries; ns, not significant; *p < .05; **p < .01; ***p < .001

OIS is primarily considered as a tumour suppressive mechanism (Collado et al., 2005), but senescent cells present in the tumour microenvironment can also drive tumour progression (Gonzalez‐Meljem, Apps, Fraser, & Martinez‐Barbera, 2018). We have previously demonstrated in mouse models of adamantinomatous craniopharyngioma (ACP), a pituitary paediatric tumour, that clusters of cells that accumulate nucleo‐cytoplasmic β‐catenin are senescent and drive tumour progression in a paracrine manner (Gonzalez‐Meljem et al., 2017). Indeed, β‐catenin‐positive cells are ki67 negative, express p21^Cip1^ and high levels of the lysosomal β‐galactosidase GLB1 (Figure S6a) (Gonzalez‐Meljem et al., 2017). To understand if GMD prodrugs could eliminate these pro‐tumourigenic senescent clusters, we used the *Hesx1^Cre/+^;Ctnnb1^lox(ex3)/+^* ACP mouse model. We have used this system before to assess the senolytic properties of cardiac glycosides (Guerrero et al., 2019, 2015). Tumoural cluster‐containing embryonic pituitaries were cultured ex vivo with vehicle or prodrug A (Figure 6a). Treatment with prodrug A preferentially eliminated the β‐catenin‐accumulating senescent cell clusters, without affecting other cell types in the pituitary such as synaptophysin + cells (Figure 6b–d). Co‐staining with an antibody recognizing cleaved caspase 3 showed that prodrug A predominantly induced apoptosis of senescent cluster cells (Figure 6e and Figure S6b). The above results suggest that GMD prodrugs could be also used to eliminate preneoplastic senescent cells.

## 3 DISCUSSION

Recently, the use of genetic mouse models in which senescent cells can be ablated has served to unveil important roles for senescence in health, disease and ageing (Baker et al., 2011, 2016; Demaria et al., 2014). Consequently, drugs have been identified that are able to phenocopy the effects of selectively eliminating senescent cells. Several of these so‐called senolytic drugs have been discovered, with Bcl2 family inhibitors such as ABT‐263 (navitoclax) and ABT‐737 (Chen et al., 2015; Yosef et al., 2016; Zhu et al., 2016) being the prototypical examples.

Here, we add galactose‐modified duocarmycin (GMD) prodrugs as a new class of senolytic agents. These GMD prodrugs are converted to their corresponding duocarmycin drugs in a manner dependent on processing by β‐galactosidase. Since senescent cells display elevated levels of lysosomal β‐galactosidase (encoded by *GLB1*), GMD selectively affects senescent cells. In this manuscript, we present evidence showing that GMD prodrugs can eliminate multiple types of senescent cells, what is consistent with SA‐β‐galactosidase being a universal marker of senescence. Our preliminary results suggest that GMD prodrugs could also be capable of eliminating bystander senescent cells arising from anticancer therapies and preneoplastic senescent cells in mouse models. This needs further investigation.

Given the promise that senolytics present for the treatment of age‐related disease, and their associated benefits over healthspan and lifespan, we believe that this study provides the basis to specifically assess the potential benefits of GMD on ageing.

Previously, the potential to harness the elevated β‐galactosidase activity of senescent cells has been exploited with galacto‐oligosaccharide encapsulated nanoparticles (GalNP) (Agostini et al., 2012). Combination of GalNP with different cargoes offers flexibility to image or eliminate senescent cells (Munoz‐Espin et al., 2018). However, this flexibility comes to the expense of having to use a modular system, comprised of both the GalNP and the cargo. Here, we propose the use of galactose‐modified prodrugs in which a single molecule (the prodrug) is sufficient to target senescent cells taking advantage of their elevated β‐galactosidase activity. While we show that duocarmycin derivatives behave as senolytic agents, this approach could be generalized to galactose‐modified prodrugs derived of other cytotoxic agents.

In summary, we have described that galactose‐modified duocarmycin prodrugs are a new class of broad‐spectrum senolytic agents. We have characterized their ability to eliminate different types of senescent cells in culture and carried out preliminary experiments in vivo. Given the increasing list of diseases that are associated with senescence, galactose‐modified duocarmycin prodrugs have the potential to be used in the context of anticancer therapies and to treat different age‐related diseases. The present study should provide the basis for investigating the potential of galactose‐modified prodrugs as senolytics to treat senescence‐associated diseases.

## 4 EXPERIMENTAL PROCEDURES

### 4.1 Drugs

The following compounds were used in this study: ABT‐263 (Selleckchem, S1001), Etoposide (Sigma‐Aldrich, E1383), Q‐VD‐OPh hydrate (Sigma‐Aldrich, SML0063), 4‐hydroxy‐tamoxifen (Sigma‐Aldrich, H7904), Doxorubicin hydrochloride (Cayman chemical, 15007). Galactose‐modified prodrugs (JHB75B, referred as prodrug A in the manuscript; JHB35B; JHB76B) and seco‐Duocarmycin analog dimer (JHB71A) were provided by Prof. Dr. L. F. Tietze. All drugs were reconstituted in DMSO unless otherwise stated.

### 4.2 Antibodies

The following primary antibodies were used in this study: mouse monoclonal anti‐BrdU (3D4; BD Biosciences, 555627), mouse monoclonal anti‐p16^INK4a^ (JC‐8; from CRUK), rabbit polyclonal anti‐β‐Catenin (Thermo Fisher Scientific, RB‐9035‐P1), mouse polyclonal anti‐p21 (BD Biosciences, 556,431), mouse monoclonal anti‐Synaptophysin (27G12; Leica, NCL‐L‐SYNAP‐299), rabbit monoclonal anticleaved caspase 3 (Asp175; 5A1E; Cell Signalling Technology, 9664), rabbit monoclonal Ki67 (Abcam, ab16667), mouse monoclonal pSer32/36‐IκBα (Cell Signalling Technology, 9246), mouse monoclonal pT2609‐DNA PKcs (Abcam, 18,356), rabbit polyclonal GLB1 (Proteintech, 15518‐1AP). We used the following secondary antibodies: goat anti‐mouse IgG (H + L, Alexa Fluor 488 conjugated, Thermo Fisher Scientific, A11029), goat anti‐mouse IgG (H + L, Alexa Fluor 594 conjugated, Thermo Fisher Scientific, A11032), goat anti‐rabbit IgG (H + L, Alexa Fluor 594 conjugated, Thermo Fisher Scientific, A11037) and goat anti‐rabbit IgG‐HRP (Santa Cruz, sc‐2004).

### 4.3 Cell lines

IMR90 cells were obtained from ATCC. IMR90 ER:RAS were generated by retroviral infection of IMR90 cells and have been described elsewhere (Banito et al., 2009; Barradas et al., 2009). IMR90 were cultured in DMEM (Gibco) supplemented with 10% foetal bovine serum (Sigma) and 1% antibiotic–antimycotic solution (Gibco). HMEC were cultured in Medium 171 (Gibco) supplemented with MEGS (Gibco), 10% foetal bovine serum (Sigma) and 1% antibiotic–antimycotic solution (Gibco). To induce OIS, IMR90 ER:RAS and HMEC ER:RAS were treated with 100 nM 4‐hydroxy‐tamoxifen (4OHT, Sigma) for 6 days. To induce chemotherapy‐induced senescence, IMR90 cells were treated with 0.5 μM Doxorubicin (Sigma) for 24 hr, or with 50 μM Etoposide (Sigma) for 48 hr (DMSO‐treatment was used as a control). To induce senescence by ionizing radiation, IMR90 were γ‐irradiated (7.5 Gy). For therapy‐induced senescence experiments (chemotherapy and irradiation), senolytic drugs were added 7 days after the induction of senescence.

### 4.4 Vector construction

pGIPZ‐based shRNA targeting *GLB1* (V3LHS_361778, V3LHS_361779, V2LHS_232389) were obtained from MRC LMS Genomics core facility.

To generate IMR90 ER:RAS‐expressing shRNAs against *GLB1*, lentiviral infections were carried out as described before (Aarts et al., 2017). Briefly, HEK293T cells were transfected with the lentiviral and packaging vectors using PEI (PEI 2500, Polysciences). Two days after transfection, HEK293T viral supernatants were collected, filtered (0.45 μM), diluted 1/4, supplemented with 4μg/ml polybrene and added to IMR90 ER:RAS cells plated the day before at a density of 1 million cells per 10‐cm dish. Four hours later, lentivirus‐containing media was replaced with fresh media. Three days after infection, cells were passaged and cultured for three days in the presence of 1μg/ml puromycin (InvivoGen) to select for infected cells.

### 4.5 BrdU incorporation

BrdU incorporation assays were performed as previously described (Georgilis et al., 2018). Briefly, for BrdU incorporation assays, the cells were incubated with 10 μM BrdU for 16–18 hr before being fixed with 4% PFA (w/v). BrdU incorporation was assessed by Immunofluorescence and High Content Analysis microscopy.

### 4.6 Immunofluorescence staining of cells

Cells were grown in 96‐well plates, fixed with 4% PFA (w/v) and stained as previously described (Georgilis et al., 2018).

### 4.7 Cytochemical SA‐β‐galactosidase assay

Cells were grown on 6‐well plates, fixed with 0.5% glutaraldehyde (w/v) (Sigma) in PBS for 10–15 min, washed with 1mM MgCl_2_/PBS (pH 6.0) and then incubated with X‐Gal staining solution (1 mg/ml X‐Gal, Thermo Fisher Scientific, 5 mM K_3_[Fe(CN)_6_] and 5 mM K_4_[Fe(CN)_6_] for 8 hr at 37°C. Bright field images of cells were taken using the DP20 digital camera attached to the Olympus CKX41 inverted light microscope. The percentage of SA‐β‐Gal‐positive cells was estimated by counting at least 100 cells per replicate sample facilitated by the “point picker” tool of ImageJ software (NIH).

For SA‐β‐galactosidase staining in tissues, frozen sections (6 μm) were fixed in ice‐cold 0.5% glutaraldehyde (w/v) solution for 15 min, washed with 1mM MgCl_2_/PBS (pH 6.0) for 5 min and then incubated with X‐Gal staining solution for 16–18 hr at 37°C as previously described (Georgilis et al., 2018). After the staining, the slides were counterstained with eosin, dehydrated, mounted and analysed by phase‐contrast microscopy. SA‐β‐Gal tissue staining was quantified using ImageJ software (NIH) by measuring the percentage of stained area in each section and multiplying it by its mean intensity value as described before (Tordella et al., 2016). To exclude the luminal spaces in the lung sections, the percentage of SA‐β‐Gal‐positive area was divided by the total lung area, as determined by eosin‐positive area using ImageJ (NIH).

### 4.8 Fluorescent SA‐β‐galactosidase assay

Cells were grown on 96‐well plates. After 8 days, cells were treated with 100 μM DDAOG (D‐6488, Thermo Fisher Scientific) 2 hr before fixation with 4% PFA for 15 min. Nuclei were stained with DAPI (1 μg/ml) for 15 min and images acquired the same day using a IN Cell Analyzer 2000 (GE Healthcare).

### 4.9 Determining senolytic activity

For oncogene‐induced senescence experiments, IMR90 ER:RAS or HMEC ER:RAS cells were plated in 96‐well dishes and induced to undergo senescence by treating them with 100 nM 4OHT for 6 days. From that point, cells were kept under serum‐starvation conditions (0.5% FBS) and 1 µM ABT‐263 or different concentrations of the galactose‐modified prodrugs were added. In parallel, the same treatments were carried out in IMR90 ER:RAS or HMEC ER:RAS cells treated with DMSO (−4OHT). These cells do not undergo senescence. Cells were fixed at day 9 after 4OHT induction and stained with DAPI (1 μg/ml) for 15 min to assess cell numbers using automated microscopy. Different models of senescence were used to test the senolytic activity of galactose‐modified prodrugs in cell culture in a similar fashion. Briefly, for therapy‐induced senescence IMR90 cells were treated with 50 μM etoposide (48 hr), 0.5 μM doxorubicin (24 hr) or left untreated, and then kept in drug‐free complete media until day 7, when the senolytics were added. Cells were fixed at day 10 after senescence induction. In all senescence types tested, 3‐day course of senolytics was applied. The percentage of cell survival was calculated dividing the number of cells after drug treatment by the number of cells treated with vehicle.

### 4.10 Co‐culture experiment

IMR90 ER:RAS (GFP positive) were plated in 96‐well plates and treated with DMSO or 4OHT for 6 days. Then, IMR90 (RFP positive) were added on top. On day 7, the cocultures were treated with different concentrations of the prodrug A or vehicle and cell survival for each population was calculated.

### 4.11 High content analysis (HCA)

IF imaging was carried out using the automated high‐throughput fluorescent microscope IN Cell Analyzer 2000 (GE Healthcare) with a 20× objective. Multiple fields within a well were acquired in order to include a minimum of 1,000 cells per sample‐well. HCA of the images was processed using the INCell Investigator 2.7.3 software as described previously (Herranz et al., 2019, 2015). Briefly, DAPI served as a nuclear mask hence allowed for the segmentation of cells with a Top‐Hat method. To detect cytoplasmic staining in cultured cells, a collar of 7–9 μm around DAPI was applied. In samples of cultured cells, a threshold for positive cells was assigned above the average intensity of unstained or negative control sample unless otherwise specified.

### 4.12 IncuCyte analysis

IMR90 ER:RAS cells were plated in 96‐well dishes and induced to undergo senescence as previously described. Different concentrations of galactose‐modified prodrug were added as normally. Cell culture media was supplemented with IncuCyte NucLight Rapid Red Reagent for cell labelling (Essen Bioscience) and IncuCyte Caspase‐3/7 reagent for apoptosis (Essen Bioscience). Four images per well were collected every 2 hr for 3 days using a 10× objective.

### 4.13 Gene expression analysis

Total RNA was extracted using Trizol reagent (Invitrogen) and the RNeasy isolation kit (Qiagen). cDNA was generated using random hexamers and SuperScript II reverse transcriptase (Invitrogen). Quantitative real‐time PCR was performed using SYBR Green PCR master mix (Applied Biosystems) in a CFX96 real‐time PCR detection system (Bio‐Rad). *Rps14* expression was used for normalization. Mouse primer pairs are:

*Cdkn1a:* CAGATCCACAGCGATATCCA*, ACGGGACCGAAGAGACAAC.*

*Cxcl1*: CTGGGATTCACCTCAAGAACATC*,* CAGGGTCAAGGCAAGCCTC*.*

*CD68:* AGGACCGCTTATAGCCCAAG, GGATGGCAGGAGAGTAACGG

*Il6:* TGATTGTATGAACAACGATGATGC, GGACTCTGGCTTTGTCTTTCTTGT.

*Rps14:* GACCAAGACCCCTGGACCT, CCCCTTTTCTTCGAGTGCTA.

### 4.14 Mouse models and drug treatments

For induction of senescence, C57BL/6J mice at 8–12 weeks of age were exposed to a sublethal dose (6 Gy) of total body irradiation. Eight weeks after, mice were injected with 50 nmols of prodrug A (i.v.) or vehicle for four consecutive days. Mice were killed 24 hr after the last injection. Mice lungs were harvested for RNA extraction, paraffin embedded for immunohistology, or frozen in OCT/Sucrose 15% (1:1) solution for cryosectioning and SA‐β‐gal stains. The mice used for all experiments were randomly assigned to control or treatment groups. Both sexes were used throughout the study.

For in vivo treatment, ABT‐263 was prepared in ethanol:polyethylene glycol 400:Phosal 50 PG at 10:30:60 as previously described (Chang et al., 2016). Mice were gavaged with vehicle (ethanol:polyethylene glycol 400:Phosal 50 PG) or ABT‐263 (50 mg/kg).

All mouse procedures were performed under licence, following UK Home Office Animals (Scientific Procedures) Act 1986 and local institutional guidelines (UCL or Imperial College ethical review committees).

### 4.15 Ex vivo culture of mouse pituitaries

Neoplastic pituitaries from 18.5dpc *Hesx1^Cre/+^;Ctnnb1^lox(ex3)/+^* embryos were dissected and placed on top of 0.2 μM Whatman filters (SLS) in 24‐well plates containing 500 μl of media (DMEM‐F12, Gibco, 1% Pen/Strep, Sigma and 1% FBS, PAA) supplemented with either prodrug A or vehicle (DMSO). Media was changed every 24h, and pituitaries were processed for analysis after 72 hr. Immunofluorescence staining was performed as previously described (Gonzalez‐Meljem et al., 2017). The proportion of β‐catenin‐accumulating and p21‐positive cells was calculated as an index out of the total DAPI‐stained nuclei. The proportion of β‐catenin‐accumulating, cleaved caspase‐3 and p21‐positive cells was calculated as an index out of the total DAPI‐stained nuclei. Over 300,000 DAPI nuclei were counted from ten histological sections per sample, in a total of twelve neoplastic pituitaries.

### 4.16 Statistical analysis

GraphPad Prism 7.0 was used for statistical analysis. Two‐tailed Student's *t* tests were used to estimate statistically significant differences between two groups. Two‐way ANOVA with Tukey's post hoc comparison was used for multiple comparisons. Values are presented as mean ± *SD* unless otherwise indicated. Asterisks (*) always indicate significant differences as follows: ns = not significant, *p < .5, **p < .01, ***p < .001, ****p < .0001.

For in vivo studies, mice were randomly assigned to treatment groups. All replicates in this study represent different mice.

## CONFLICT OF INTEREST

J.G. owns equity and has acted as a consultant for Unity Biotechnology and Geras Bio. J.G., A.G. and N.H. are named inventors in an MRC patent related to senolytic therapies (not related to the work presented here).

## AUTHORS’ CONTRIBUTION

A.G. designed performed and analysed the cell culture experiments and wrote the first draft of the manuscript. R.G. designed performed and analysed the experiments with the ACP model. N.H. designed, performed and analysed the in vivo experiments. L.F.T. designed and synthetized the duocarmycin derivatives and secured funding. A.U., J.P. M.‐B. and D.J.W. designed the in vivo experiments and secured funding. J.G. conceived and designed the project, secured funding and wrote the manuscript, with all authors providing feedback.

## Supporting information

Fig S1‐S6

## Data Availability

The data that support the findings of this study are available from the corresponding author upon reasonable request.
