# Supplementary material for: Galactose‐modified duocarmycin prodrugs as senolytics
Source: Aging Cell. 2020 Mar 16;19(4):e13133. doi: 10.1111/acel.13133 (PMC7189988; doi:10.1111/acel.13133)
Supplement: Supplementary file 1 — Fig S1‐S6 [file ACEL-19-e13133-s001.pdf]

SUPPORTING INFORMATION for

Galactose-modified duocarmycin prodrugs as senolytics

Ana Guerrero, Romain Guiho, Nicolás Herranz, Anthony Uren, Dominic J. Withers,

Juan Pedro Martínez-Barbera, Lutz F. Tietze and Jesús Gil

Supplemental information includes 6 supplementary figures and their legends.

a

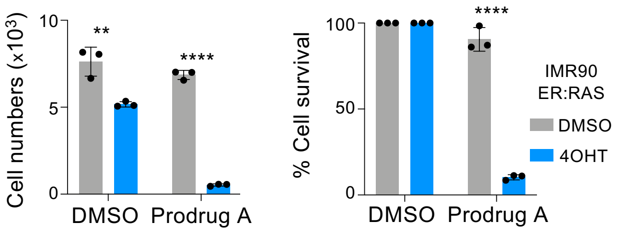

b

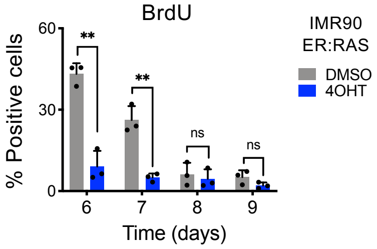

Supplementary Figure S1. Prodrug A selectively kills senescent cells (a) Quantification of survival after treatment with prodrug A or vehicle in IMR90 ER:RAS cells. Left panel, cell numbers. Right panel, cell survival normalised at their respective vehicle (DMSO)-treated cells, expressed as percentages. **(b)** Senescent cells are arrested at the time of treatment with duocarmycin and duocarmycin derivatives. Quantification of immunofluorescence staining for BrdU. IMR90 ER:RAS were treated with 4-OHT or vehicle (DMSO) for 6 days to induce senescence. Cell were then fixed (day 6) or kept under serum-starvation (0.5% FBS) conditions and fixed 24h (day 7), 48h (day 8) or 72h (day 9) later to assess BrdU incorporation. A 16 h pulse of BrdU was given before fixation (n = 3). All statistical significances were calculated using unpaired two-tailed Student's t-tests. Error bars represent mean ± s.d; n represents independent experiments.; ns, not significant; *$P < 0.05$; **$P < 0.01$; ***$P < 0.001$, ****$P < 0.0001$.

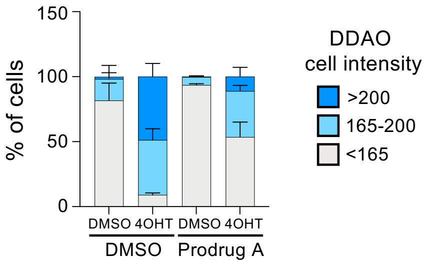

Supplementary Figure S2. Treatment with prodrug A kill senescent cells with high and very high SA-β-galactosidase activity. Cells were divided in 3 groups based on their SA-β-galactosidase activity (low activity, cell intensity < 165; high activity, cell intensity values between 165-200; very high, cell intensity > 200) (*n* = 3).

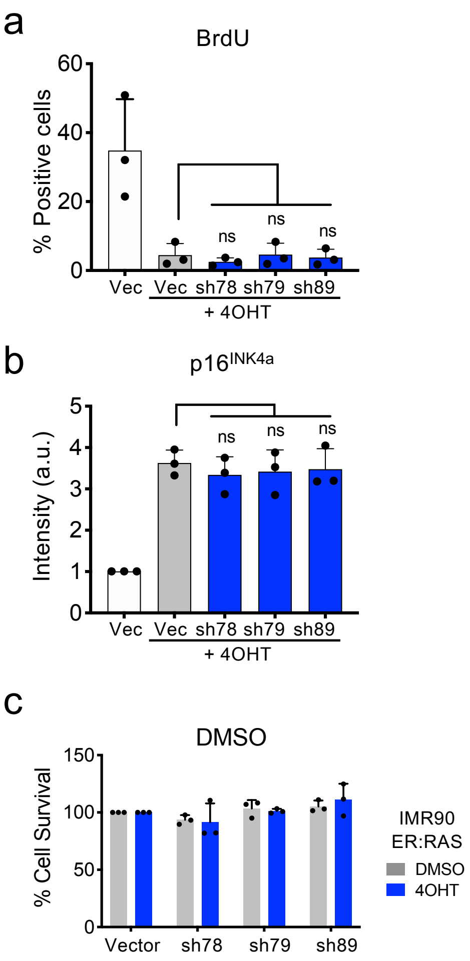

Supplementary Figure S3. Characterization of IMR90 ER:RAS cells with reduced GLB1. (a-b) IMR90 ER:RAS cells with reduced β-galactosidase expression undergo senescence as assessed by BrdU incorporation (**a**) and p16^{INK4a} staining (**b**) (*n* = 3). Statistical significance was calculated using one-way ANOVA. (**c**) Quantification of cell survival of senescent and control IMR90 ER:RAS infected with different shRNAs targeting *GLB1* or an empty vector and treated with vehicle (DMSO) for 3 days (*n* = 3). Statistical significance was calculated using two-tailed, Student's *t*-test. All error bars represent mean ± s.d; *n* represents independent experiments; ns, not significant; *P < 0.05; **P < 0.01; ***P < 0.001.

Guerrero et al. **Sup. Fig. S4**

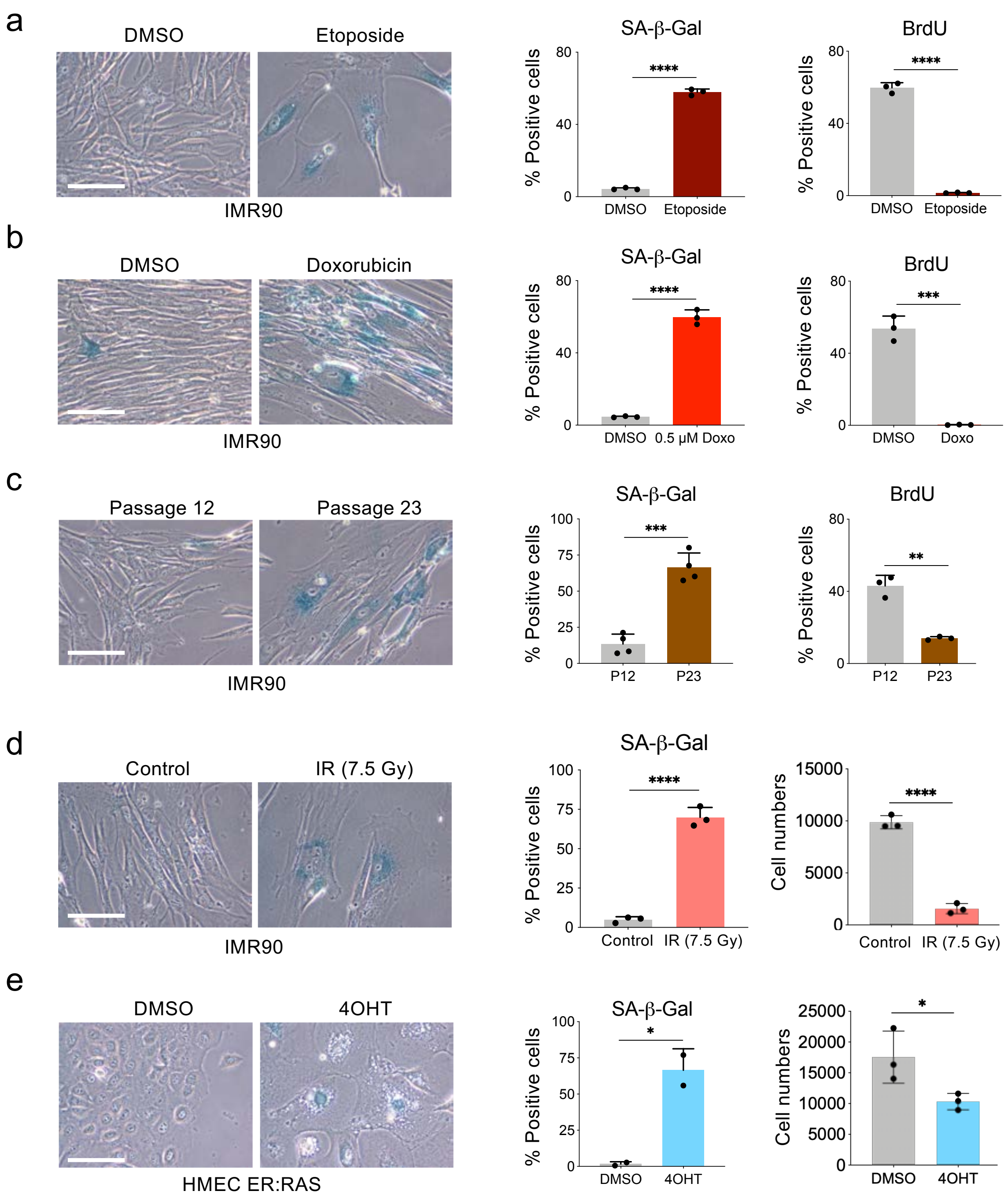

Supplementary Figure S4. Senescence-associated β-galactosidase activity across different types of senescent cells. Representative images (left) and quantification of SA-β-galactosidase activity and BrdU incorporation (right) in **(a)** etoposide-induced senescence, **(b)** doxorubicin-induced senescence and **(c)** replicative senescence. Representative images (left) and quantification of SA-β-galactosidase activity and cell numbers (right) in **(d)** irradiation-induced senescence and oncogene-induced senescence in HMEC ER:RAS **(e)**. All statistical significances were calculated using two-tailed, Student's *t*-test. All error bars represent mean ± s.d; *n* represents independent experiments; ns, not significant; *P < 0.05; **P < 0.01; ***P < 0.001; ***P < 0.0001.

a

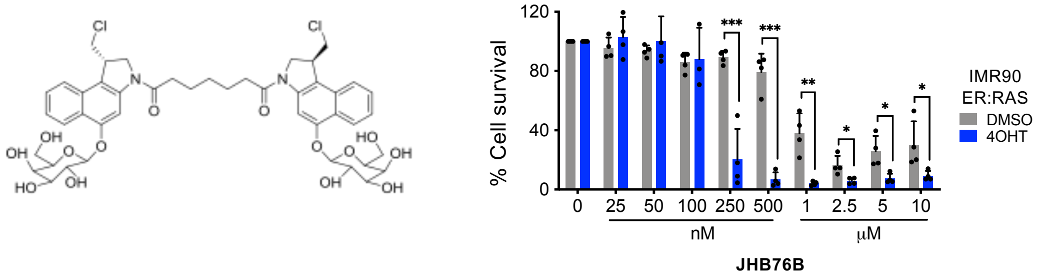

b

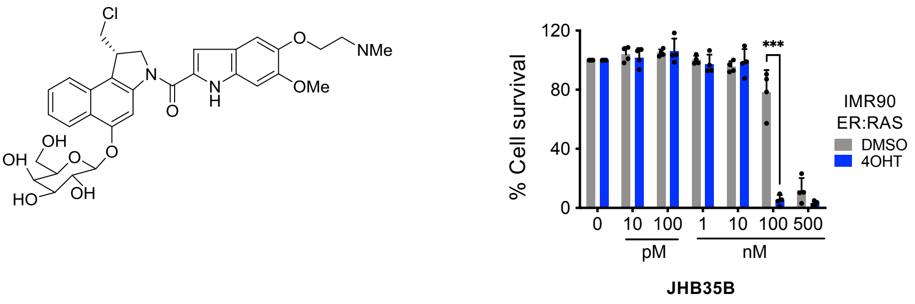

Supplementary Figure S5. Galactose-modified duocarmycin derivatives selectively kill senescent cells. Quantification of cell survival after treatment with the galactose-modified duocarmycin derivatives, JHB76B **(a)** and JHB35B **(b)** in the context of oncogene-induced senescence in IMR90 ER:RAS (*n* = 4). A reduced version of this graph is shown in Figure 3f. All statistical significances were calculated using unpaired two-tailed Student's *t*-tests. All error bars represent mean ± s.d; *n* represents independent experiments.; ns, not significant; *$P < 0.05$; **$P < 0.01$; ***$P < 0.001$.

Guerrero et al. **Sup. Fig. S6**

a

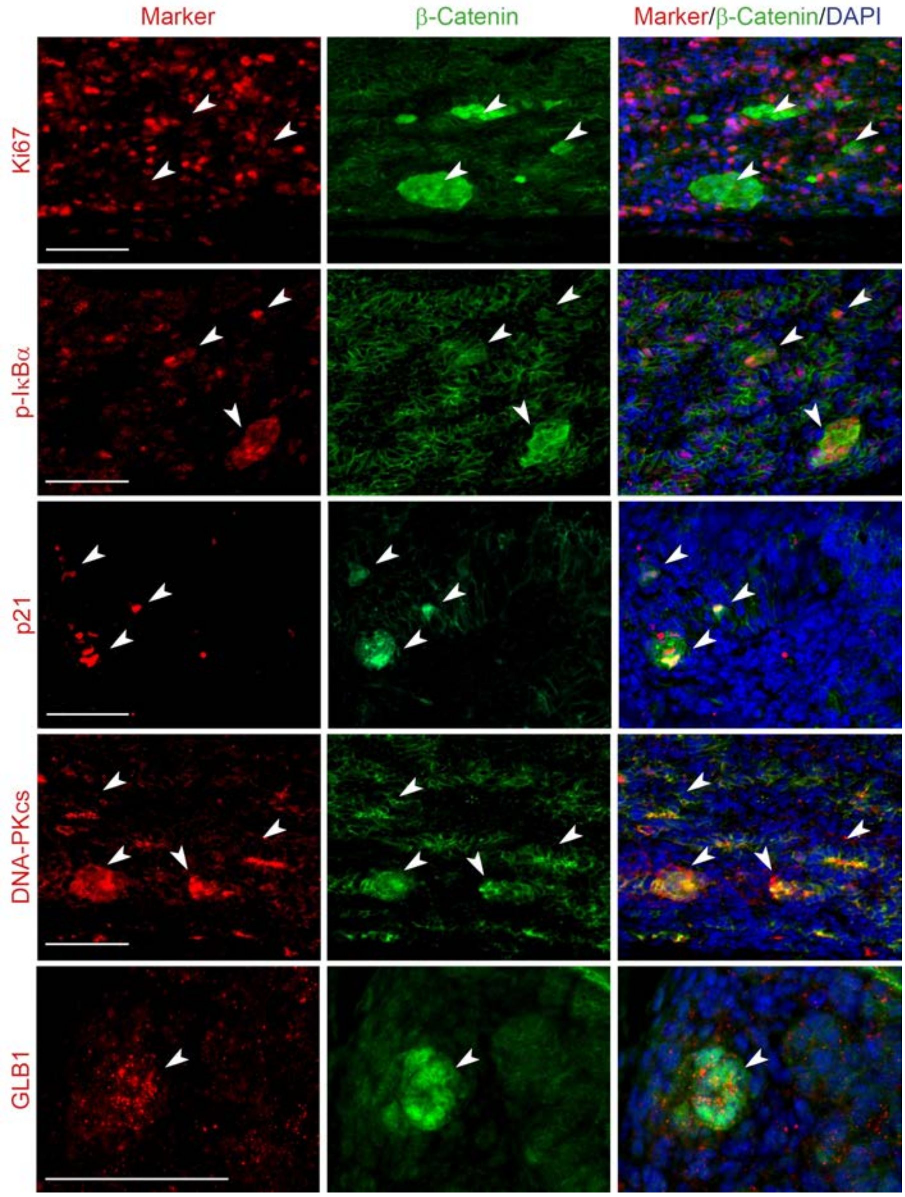

b

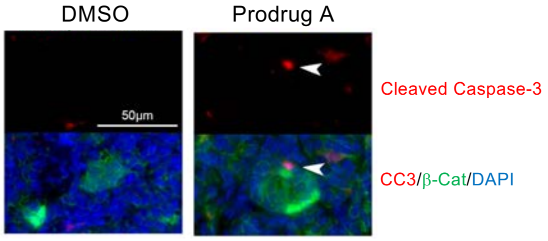

Supplementary Figure S6. Clusters of β-catenin-accumulating cells in mouse embryonic pituitaries are senescent. (a) Representative pictures of β-catenin-positive cells (green) co-stained with different markers of senescence (red). **(b)** Galactose-modified duocarmycin derivatives induce apoptosis of β-catenin-accumulating preneoplastic cells in a model of ACP. Representative pictures of pituitaries treated with DMSO (left) or prodrug A (right). Top panels show cleaved caspase-3 staining (red). Bottom panels show a merged composite image showing cleaved caspase-3 staining (red), β-Catenin (green) and DAPI (blue). Scale bar, 50 μM.
